# Impact of the rs1024611 Polymorphism of *CCL2* on the Pathophysiology and Outcome of Primary Myelofibrosis

**DOI:** 10.3390/cancers13112552

**Published:** 2021-05-22

**Authors:** Elena Masselli, Cecilia Carubbi, Giulia Pozzi, Antonio Percesepe, Rita Campanelli, Laura Villani, Giuliana Gobbi, Sabrina Bonomini, Giovanni Roti, Vittorio Rosti, Margherita Massa, Giovanni Barosi, Marco Vitale

**Affiliations:** 1 Department of Medicine and Surgery, Anatomy Unit, University of Parma, 43126 Parma, Italy; elena.masselli@unipr.it (E.M.); cecilia.carubbi@unipr.it (C.C.); giulia.pozzi@unipr.it (G.P.); giuliana.gobbi@unipr.it (G.G.); 2 Department of Medicine and Surgery, Genetics Unit, University of Parma, 43126 Parma, Italy; antonio.percesepe@unipr.it; 3 Center for the Study of Myelofibrosis, Biochemistry, Biotechnology and Advanced Diagnostics Laboratory, IRCCS Policlinico S. Matteo Foundation, 27100 Pavia, Italy; r.campanelli@smatteo.pv.it (R.C.); l.villani@smatteo.pv.it (L.V.); v.rosti@smatteo.pv.it (V.R.); barosig@smatteo.pv.it (G.B.); 4 Hematology Unit, University Hospital of Parma, 43126 Parma, Italy; sbonomini@ao.pr.it; 5 Department of Medicine and Surgery, Hematology Unit, University of Parma, 43126 Parma, Italy; giovanni.roti@unipr.it; 6 Biochemistry, Biotechnology and Advanced Diagnostics Laboratory, IRCCS Policlinico S. Matteo Foundation, 27100 Pavia, Italy; m.massa@smatteo.pv.it

**Keywords:** primary myelofibrosis, myeloproliferative neoplasms, single nucleotide polymorphisms, CCL2, CCR2, Akt

## Abstract

**Simple Summary:**

Among myeloproliferative neoplasms, primary myelofibrosis (PMF) is considered the paradigm of inflammation-related cancer development. Host genetic variants such as single nucleotide polymorphisms (SNPs) can affect cytokine/chemokine gene expression and may therefore have a role in a disease with a strong inflammatory component such as PMF. Here we demonstrate that the homozygosity for the rs1024611 SNP of the chemokine CCL2 represents a high-risk variant and a novel host genetic determinant of reduced survival in PMF, providing opportunities for *CCL2* SNP genotyping as a potential novel strategy to risk-stratify patients. The rs1024611 genotype also influences CCL2 production in PMF cells, which are electively sensitive to CCL2 effects because of their unique expression of its receptor CCR2. Finally, ruxolitinib is capable of effectively down-regulating CCR2 expression, de-sensitizing PMF cells to the IL-1β-dependent pro-inflammatory stimulus.

**Abstract:**

Single nucleotide polymorphisms (SNPs) can modify the individual pro-inflammatory background and may therefore have relevant implications in the MPN setting, typified by aberrant cytokine production. In a cohort of 773 primary myelofibrosis (PMF), we determined the contribution of the rs1024611 SNP of CCL2—one of the most potent immunomodulatory chemokines—to the clinical and biological characteristics of the disease, demonstrating that male subjects carrying the homozygous genotype G/G had an increased risk of PMF and that, among PMF patients, the G/G genotype is an independent prognostic factor for reduced overall survival. Functional characterization of the SNP and the CCL2-CCR2 axis in PMF showed that i) homozygous PMF cells are the highest chemokine producers as compared to the other genotypes; ii) PMF CD34+ cells are a selective target of CCL2, since they uniquely express CCR2 (CCL2 receptor); iii) activation of the CCL2-CCR2 axis boosts pro-survival signals induced by driver mutations via Akt phosphorylation; iv) ruxolitinib effectively counteracts CCL2 production and down-regulates CCR2 expression in PMF cells. In conclusion, the identification of the role of the CCL2/CCR2 chemokine system in PMF adds a novel element to the pathophysiological picture of the disease, with clinical and therapeutic implications.

## 1. Introduction

Myelofibrosis (MF) is the most aggressive Philadelphia-chromosome negative myeloproliferative neoplasm (MPN), typified by abnormal proliferation and differentiation of hematopoietic progenitors, variable degree of bone marrow fibrosis and cytopenias, elevated circulating CD34^+^ cells, splenomegaly and risk of blast transformation (BT) [1,2]. MF represents the paradigm of onco-inflammatory disorders: chronic inflammation, fueled by the malignant hematopoietic clone itself, contributes to systemic manifestations and elicits clonal evolution, in a self-perpetrating vicious cycle [3,4,5]. In fact, it is clearly emerging that while the onset of the disease is caused by acquired somatic mutations in target myeloid genes, its progression is driven, at least in part, by inflammation [6]. Dysregulation of the JAK/STAT pathway, resulting from the occurrence of one of the three so called “driver mutations”, i.e., *JAK2* exon 14 and exon 12 mutations, thrombopoietin receptor gene (Myeloproliferative Leukemia Protein, MPL) mutations or calreticulin (CALR) mutations, has been traditionally identified as the link between the neoplastic clone and cytokine overproduction in MF (and MPN in general) [7].

Host genetic variants such as single nucleotide polymorphisms (SNPs) are emerging as important players in modulating the individual pro-inflammatory background by affecting cytokine/chemokine gene expression and mRNA levels [8]. Indeed, inherited genetic variants predisposing to MF onset, phenotype and outcome, such as the *JAK2* GGCC_46/1 haplotype [9,10] and the rs6198 SNP of *NR3C1* [11], have also the potential to predispose to enhanced inflammatory state and autoimmune disorders [12,13,14].

CCL2 (the systematic designation for the Monocyte Chemoattractant Protein-1, MCP-1) is a member of the C-C class of the beta chemokine family and one of the most potent immunomodulatory cytokines known to be elevated in MF. *CCL2* expression is regulated at the transcriptional level by a complex network of transcription factors, including NF-kB, C/EBP, AP-1 and Sp-1 [15]. Transcriptional events lead to CCL2 secretion upon inflammatory noxa by a variety of cell types, such as monocytes, fibroblasts, endothelial cells, vascular smooth muscle cells and T cells.

CCL2 exerts its effects by preferentially engaging its cognate receptor CCR2 [16,17], expressed on the surface of target cells. The activation of CCR2 by CCL2 induces the activation of downstream signaling pathways including G-proteins, the mitogen-activated protein kinase/extracellular signal–regulated kinase (MAPK/ERK) pathway, the phosphoinositide 3-kinase/protein kinase B (PI3K/AKT) pathway and the Janus kinase/signal transducer and activator of transcription (JAK/STAT) pathway [18]. Besides triggering immune cell trafficking into the sites of inflammation, the activation of the CCL2-CCR2 axis can promote both direct and indirect pro-tumorigenic effects, fostering tumor cell proliferation, stemness and survival, as well as neoangiogenesis, local invasiveness and metastasis [19]. Consistently, CCL2 is overexpressed by a variety of solid and hematologic neoplasms, particularly breast, prostate, gastric, colorectal, pancreatic cancers and non-Hodgkin lymphomas [20].

In the general population, CCL2 expression levels display a significant inter-individual variability, and this has been ascribed to SNPs in the regulatory regions of *CCL2* gene. Specifically, an A to G substitution in the distal regulatory region of the *CCL2* gene at position −2518 from the transcription start site (subsequently annotated as the rs1024611 SNP) influences the transcriptional activity of *CCL2* due to a mechanism of allelic expression imbalance and preferential transcription of the G allele. As a result, the functional effect of the rs1024611 SNP on CCL2 expression is dose-dependent, with cells from homozygous individuals producing more chemokine than cells from heterozygous individuals [21,22,23]. The prevalence of high CCL2-producing genotypes has been associated with increased susceptibility to several chronic inflammatory conditions including autoimmune disorders [8] and increased risk of cancer in Caucasian individuals, as documented by a recent meta-analysis involving 1,4617 subjects [24].

We recently demonstrated that patients with post-polycythemia vera/essential thrombocythemia MF are enriched in the polymorphic allele variant of the rs1024611 SNP of *CCL2*, and its presence correlates with adverse clinical features [25]. Moving from this finding, we examined how *CCL2* rs1024611 allele variants impact the baseline phenotype and disease progression in a cohort of 773 primary MF (PMF). We approached this endeavor by using a data-base of patients with long duration of follow-ups, low rates of missing outcome data, and adjustment for potential confounders. We then proved the functional relevance of our findings in PMF pathophysiology by: (i) demonstrating that rs1024611 genotypes affect CCL2 production in PMF cells, (ii) assessing the effects of CCL2/CCR2 axis activation PMF hematopoietic progenitors, (iii) testing the down-regulation of the CCL2-CCR2 chemokine system by immunomodulatory therapy with ruxolitinib.

## 2. Materials and Methods

### 2.1. Subjects

The protocols were approved by the ethical committees of the institutions (Center for the Study of Myelofibrosis, IRCCS Policlinico S. Matteo, Pavia, Italy and Parma University Hospital, Parma, Italy, Prot. 11537-14/03/2019 and 18698-03/05/2019); all patients signed a written consent form.

The cohort for genotype-phenotype correlation studies consisted of 773 Caucasian PMF patients consecutively enrolled at the Center for the Study of Myelofibrosis, IRCCS Policlinico S. Matteo, from 1990 to December 2019. DNA from 323 Caucasian control subjects was provided by the Unit of Medical Genetics, University Hospital of Parma.

For functional in vitro studies, 33 PMF, 6 ET, 6 PV and 7 granulocyte colony stimulating factor-mobilized donors (healthy donors, HD) were recruited at the Hematology and BMT Unit of Parma University Hospital (from September 2016 to January 2021).

The diagnosis of ET, PV, pre- and overtPMF was formulated/revised according to the 2016 WHO classification. Clinical, biological and histopathological data were collected from patients’ records and listed in the Supplementary Materials. PCR analysis and Next Generation Sequencing (see the Supplementary Materials) were used to detect mutations in selected myeloid genes, including driver mutations in *JAK2*, *CALR*, *MPL* and high molecular risk (HMR) mutations in *EZH2*, *ASXL1*, *IDH1/IDH2* and *SRSF2*, previously shown to be prognostically informative in PMF [26].

For in vitro studies, patients’ and HD peripheral blood/bone marrow samples (~30 mL and 15 mL, respectively, collected in ethylenediaminetetraacetic acid tubes) were utilized for mononuclear cells (MNCs) isolation, CD34+ cell purification and in vitro cell treatments as subsequently described. Sampling was performed at the time of diagnosis, and all patients were off cytoreductive treatment; for specific experiments, 8 PMF were sampled before and after ruxolitinib start.

### 2.2. Genotype Analysis

DNA was extracted by PureLink^®^ Genomic DNA Kit (Invitrogen, Waltham, MA, USA, cat. N. K182002) from 200 µL of whole blood, and *CCL2* rs1024611 SNP genotyping was performed by TaqMan^®^ Predesigned SNP Genotyping Assays (Applied Biosystems, Foster City, CA, USA) as already described [25].

### 2.3. Cell Cultures

Peripheral blood MNCs obtained by a Ficoll–Hypaque gradient from n. 25 therapy naïve PMF stratified according to the *CCL2* rs1024611, and from n. 6 PMF before (T0) and after ruxolitinib start (at 1, 3 and 9 months of therapy—T1, T2 and T3), were in part pelleted (basal) and in part seeded at the optimal density of 1 × 10^6^ cells/mL in 10% fetal bovine serum-enriched RPMI medium with 1.1 mg/mL of IL1-β for 20 h, as described [21]. Cells were then pelleted and subjected to subsequent treatments to assess CCL2 expression.

Primary CD34+ cells were isolated from n. 16 therapy naïve PMF, from n. 6 PMF before and after ruxolitinib start, from n. 6 ET, n. 6 PV and n. 7 HD by immunomagnetic positive selection, as described [27]. Only samples exceeding 95% purity were used for subsequent experiments. CCR2 expression was assessed by flow cytometry (CytoFLEX, Beckman Coulter, Brea, CA, USA) labeling cells with anti-CCR2-PE (R&D Systems, Minneapolis, MN, USA, cat. N. FAB151P), immediately after isolation and, from representative ET/PV cases, after 48 h of culture in serum-free X-vivo medium supplemented with 3 ng/mL IL-3, 50 ng/mL SCF and 200 ng/mL TPO (PeproTech, Rocky Hill, NJ, USA, cat. N. 200-03, 300-07 and 300-18), alone or in combination with: (i) rhCCL2 100 ng/mL; (ii) IL-1β 1.1 ng/mL and TNFα 10 ng/mL (PeproTech, cat. N. 200-01B and N. 300-01A); (iii) rhCCL2, IL-1β and TNFα.

To test signal transduction activation, CD34+ cells from n. 7 PMF and n.3 HD were cultured up to 24 h in serum-free X-vivo medium with and without 100 ng/mL of rhCCL2 (R&D Systems, Minneapolis, MN, USA, cat. N. 279-MC). The cells were then pelleted and subjected to western blot analysis.

A *JAK2*V617F-positive human erythroleukemia cell line (HEL) was grown in 10% fetal bovine serum-enriched RPMI medium at the optimal density of 0.5 × 10^6^ cells/mL, alone and supplemented with: (i) rhCCL2 100 ng/mL; (ii) IL-1β 1.1 ng/mL and TNFα 10 ng/mL; (iii) rhCCL2, IL-1β and TNFα. Before treatment and after 48 h of culture, the cells were checked for CCR2 expression by flow cytometry, as described above.

### 2.4. Real-Time Quantitative RT-PCR (qRT-PCR)

To test CCL2 expression, RNA was extracted by the RNeasy Mini Kit (Qiagen, Hilden, Germany, cat. N. 74106) from aliquots (~1.5 × 10^6^) of cells obtained as described above. One microgram of RNA was reverse-transcribed using the High-Capacity RNA-to-cDNA kit (Applied Biosystems, Foster City, CA, USA, cat. N. 4387406) and then subjected to real time PCR using PowerUp™ SYBR™ Green Master Mix (Applied Biosystems, cat. N. A25742). The cDNAs were amplified with specific primers for *CCL2* (fw, 5′-CATAGCAGCCACCTTCATTCC-3′, rv 5′-TCTCCTTGGCCACAATGGTC-3′) as described in [28]. Glyceraldehyde 3-phosphate dehydrogenase, *GAPDH* (fw, 5′- TTGAGGTCAATGAAGGGGTC -3′, rv 5′- GAAGGTGAAGGTCGGAGTCA -3′), was used as the housekeeping gene control. All reactions were run in triplicate. A semi-quantitative analysis was based on the cycle number (CT) at which the SYBR Green fluorescent signal crossed a threshold in the log-linear range. The fold-change in CCL2 mRNA expression after IL-1β stimulation was compared with untreated, freshly pelleted cells and is shown relative to the change in the expression of GAPDH mRNA (internal control) [(2^-[(*C*T CCL2-*C*T GAPDH)IL1-β − (*C*T CCL2-*C*T GAPDH)untr^, relative 2^−ΔΔ*C*T^ values].

### 2.5. Western Blotting

Fifty micrograms of proteins were separated on 14% (for CCL2 detection) and 10% (for signaling pathway analysis) SDS-polyacrylamide gel electrophoresis, transferred to a nitrocellulose membrane and incubated with anti-CCL2 (Thermo Fisher Scientific, Waltham, MA, USA, cat. N. MA5-17040), anti-JAK2 (Cell Signaling Technology, Danvers, MA, USA, cat. N. 3230), anti-phophoJAK2 (Tyr 1007/1008) (Cell Signaling Technology, cat. N. 3771), anti-STAT5 (Cell Signaling Technology, cat. N. 9358), anti-phosphoSTAT5 (Tyr 694) (Cell Signaling Technology, cat. N. 9314), anti-Akt (Cell Signaling Technology, cat. N. 9272), anti-phopshoAkt (Ser 473) (Cell Signaling Technology, cat. N. 9271), anti-P44/42 ERK1/2 (Cell Signaling Technology, cat. N. 4695), anti-phosphoP44/42 ERK1/2 (Thr202/Tyr 204) (Cell Signaling Technology, cat. N. 9101) and anti-GAPDH (Merck Millipore, Burlington, MA, USA, cat. N. MAB374). Data are expressed as relative western blot optical density (O.D.) values using GAPDH as loading control protein. For specific calculations, please refer to the figures’ legends.

### 2.6. Statistical Analysis

*CCL2* risk groups were defined according to the number of copies (0, 1 or 2) of the risk allele (G allele). After verifying that PMF with only 1 copy of the risk allele (A/G), had a similar risk of the reference group with 0 copies (A/A), we used a recessive genetic model to compare PMF with 2 copies of the risk variant (G/G) vs. A/A and A/G PMF. Genotype-phenotype correlations with demographic characteristics and disease parameters at the time of diagnosis were performed by χ^2^/Fisher exact or Kruskal–Wallis tests, as applicable.

The association between *CCL2* risk groups and patients’ hematological outcomes was assessed by Cox proportional-hazards models. Specifically, we analyzed the risk of incurring into severe anemia (<100 g/L), massive splenomegaly (>10 cm below left costal margin), leukocytosis (>12 × 10^9^/L) and blats transformation. Data were censored at the time of therapy or last follow-up. Overall survival was estimated using a Kaplan–Meier product limit estimator. Multivariate analysis was also performed including well-established clinical prognostic factors (IPSS score parameters). Differences between the actuarial estimates were tested using the log-rank test. Analyses were performed with STATISTICA© software (DELL, Round Rock, TX, USA, release 13.1). For in vitro experiments, data were presented as mean ± SEM of independent experiments. Statistical analyses were performed by a one-way ANOVA followed by a Tukey test or *t*-test, as applicable. GraphPad Prism software (GraphPad Software, San Diego, CA, USA) was used for all calculations.

## 3. Results

### 3.1. Male Subjects Homozygous for the rs1024611 SNP of CCL2 Had an Increased Risk of PMF

Case and control groups were aligned for age and gender distribution. Genotypic frequencies were in Hardy–Weinberg equilibrium both in PMF and CTRL cohort (*p* > 0.05), consistently with our previous report [25].

When considering the overall PMF population, the *CCL2* SNP does not emerge as a major host predisposition factor for PMF. In fact, consistently with our previous report [25], the *CCL2* rs1024611 high-risk variant, tagged by the G allele, occurred as frequently in PMF (400/1546 alleles; 25.9%) as in CTRL (152/646 alleles, 23.5%; OR, 1.13). Genotypic frequencies in PMF and CTRL were similar as well (PMF: A/A: 55.6%, A/G: 37.0%, G/G 7.4%; CTRL: A/A 57.6%, A/G 37.8%, G/G 4.6%, Table 1).

To further analyze the relationship between *CCL2* SNP and PMF risk, we performed a stratified analysis by gender and age (Table 1). When the entire study cohort (PMF and CTRL) was stratified by gender, male subjects carrying two copies of the G allele showed a higher risk of PMF than those with zero or one copy (OR, 2.52, 95% CI, 1.11–5.72, *p* = 0.022). Additionally, the PMF male cohort was significantly enriched in G/G subjects (43/464, 9.3%) as compared to the PMF female cohort (14/309, 4.5%, OR = 2.15, 95% CI, 1.15–4.00, *p* = 0.014); G-allele frequency was significantly higher as well (28% vs. 22.7%, OR = 1.33, 95% CI, 1.05–1.68, *p* = 0.02). By contrast, no differences in the distribution of genotypic and allelic frequencies were detected among the PMF- and CTRL- female cohorts (Table 1). These data suggest a potential genetic interplay between gender and the rs1024611 G-allele variant, affecting the risk of PMF.

Although the overall patient cohort was characterized by relatively young subjects (median age 50.7 years, range 6–83), PMF homozygous for the SNP were slightly older at the time of diagnosis (median 54.4 years, range 18–83) as compared to A/A and A/G (median 50.4 years, range 6–81, *p* = 0.048, Table 1), in line with our previous findings indicating that age ≥52 years is associated with a higher inflammatory burden [29].

### 3.2. Homozygosity for the rs1024611 SNP of CCL2 Correlates with Reduced Survival in PMF

We assessed whether the homozygosity for the SNP clustered with specific clinical and biological features, analyzing the distribution of hematological characteristics at the time of disease onset in G/G PMF as compared to the other genotypes (A/A + A/G). No association was found by comparing the baseline clinical parameters of G/G vs. A/A + A/G PMF, distribution of *JAK2V617F*, *MPLW515* and *CALR* mutations were similar in the groups. Triple-negative patients were also distributed equally. Specularly, G/G patients were not enriched in HMR mutations. (Supplementary Materials Table S1). Overall, these data indicate that PMF homozygous for the rs1024611 SNP of *CCL2* display hematological characteristics similar to those of heterozygous and wild types.

We then investigated whether the homozygosity for the SNP could impact the disease outcome, assessing whether PMF harboring the G/G genotype have a higher chance of incurring in: (i) severe anemia (Hb < 100 g/L), massive splenomegaly (≥10 cm below costal margin) or leukocytosis (WBC ≥ 12 × 10^9^/L); (ii) blast transformation (defined as a proportion of >20% blasts in the peripheral blood or bone marrow according to the IWG-MRT criteria [30]); (iii) death for any cause (overall survival). On the basis of the recessive genetic model, the hazard ratio (HR) to develop anemia, massive splenomegaly or leukocytosis or to incur into blast transformation was similar in the G/G group as compared to A/A+A/G (HR, 1.31, *p* = 0.183; HR, 1.03, *p* = 0.927; HR, 1.19, *p* = 0.438 and HR, 1.07, *p* = 0.849, respectively).

**Figure 1:**
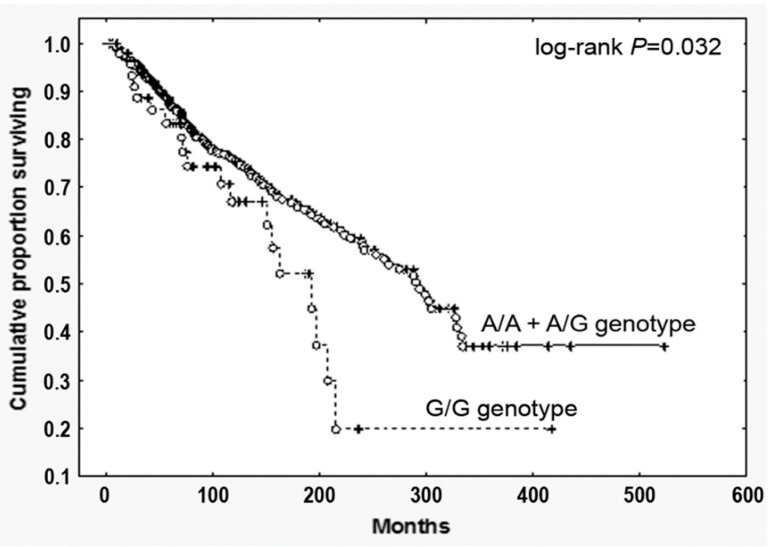
Overall survival of PMF patients stratified according to the *CCL2* rs1024611 SNP. Cumulative survival (Kaplan Meier) estimates during follow-up of 773 genotyped PMF, stratified according to the *CCL2* rs1024611 allele variants (G/G vs. A/A+A/G), *p* = 0.032.

By contrast—and most relevantly—PMF harboring the G/G genotype displayed a significantly reduced survival (HR, 1.69, 95% CI, 1.05–2.70; *p* = 0.032, Figure 1). To test the prognostic accuracy of the homozygosity for the SNP to predict survival, we performed a multivariate analysis combing the G/G genotype with other well-established clinical prognostic parameters included in the IPSS scoring system (age > 65 years, Hb < 100 g/L, peripheral blasts ≥1, constitutional symptoms, WBC > 25 × 10^9^/L). We found that the homozygosity for the SNP showed a significant correlation in both univariate and multivariate analysis (Supplementary Materials Table S2), holding therefore an independent prognostic value in predicting survival in PMF.

### 3.3. Homozygosity for the rs1024611 SNP of CCL2 Accounts for Higher Chemokine Production in PMF

**Figure 2:**
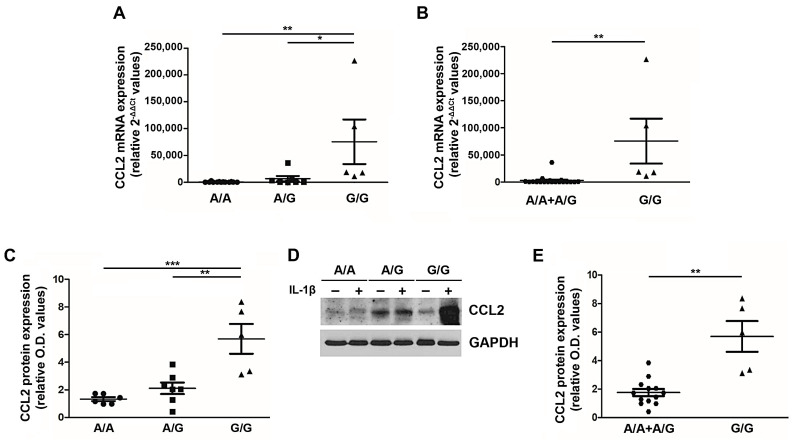
CCL2 expression by PMF MNCs according to the rs1024611 SNP. Fold-changes (2-[(CT CCL2-CT GAPDH)IL1-β − (CT CCL2-CT GAPDH)untr, relative 2-ΔΔCT values) in CCL2 mRNA expression upon ex vivo IL1-β stimulation of MNCs from PMF patients stratified according to the rs1024611 genotype: A/A (n. 13) vs. A/G (n. 7) vs. G/G (n.5) (**A**) and A/A + A/G (n. 20) vs. G/G (n. 5) (**B**). Data are expressed as mean ± SEM. * *p* < 0.05, ** *p* < 0.01 by one-way ANOVA followed by Tukey test (**A**) or *t*-test (**B**). Fold-changes [(O.D.CCL2/O.D.GAPDH)IL-1β/[(O.D.CCL2/O.D.GAPDH)UNTR, relative western blot optical density (O.D.) values] in CCL2 protein expression upon ex vivo IL1-β stimulation of MNCs from PMF patients stratified according to the rs1024611 genotype A/A, n. 6 vs. A/G, n. 7 vs. G/G, n.5 (**C**) and A/A + A/G, n. 13 vs. G/G n. 5 (**E**). Data are expressed as mean ± SEM. ** *p* < 0.01, *** *p* < 0.001 by ANOVA followed by Tukey test (**C**) or *t*-test (**E**). (**D**) Western blot showing CCL2 protein expression in MNCs from a representative A/A, A/G and G/G PMF, at a basal state (−) and upon IL1-β stimulation (+).

Given the clinical impact of the G/G genotype on the PMF patient outcome, we sought to understand the functional relevance of this SNP in PMF cells. Peripheral blood MNCs from PMF patients stratified according to the three genotypes (wild type A/A, heterozygous A/G and homozygous G/G) were stimulated ex vivo with 1.1 ng/mL of IL-1β [21], and after 20 h CCL2 expression (mRNA and protein) was assessed (Figure 2). The G allele exerts a dose-dependent effect on CCL2 expression, with G/G PMF significantly overexpressing CCL2 transcript (relative 2^−ΔΔCT^ values: 75,564.1 ± 41,453.6) and protein (relative O.D. values: 5.69 ± 1.08) as compared to A/A (relative 2^−ΔΔCT^ values: 571.1 ± 213.6, *p* < 0.01; relative O.D. values: 1.33 ± 0.14, *p* < 0.001) and A/G (relative 2^−ΔΔCT^ values: 6,770.2 ± 4,964.9, *p* < 0.05; relative O.D. values: 2.11 ± 0.41, *p* < 0.01) genotypes (Figure 2A,C,D). By adopting the same recessive genetic model used for genotype-phenotype association, we show that G/G PMF significantly overexpress CCL2 transcript and protein as compared to A/A + A/G subjects (relative 2^−ΔΔCT^ values: 2,740.8 ± 1,789,8, *p* < 0.01; relative O.D. values: 1.75 ± 0.26, *p* < 0.01, Figure 2B,E). These data prove that the rs1024611 SNP of *CCL2* is functionally relevant in PMF cells, and pinpoint G/G PMF as the highest chemokine-producer disease category.

### 3.4. CCL2-Receptor CCR2 Is Uniquely Expressed by PMF Hematopoietic Progenitors 

We then asked whether PMF hematopoietic progenitors could be a target of CCL2 by testing the expression of its primary receptor CCR2 on PMF CD34+ and comparing it with CCR2 expression on CD34+ cells from HD, PV/ET and *JAK2*V617F^pos^ HEL cells.

**Figure 3:**
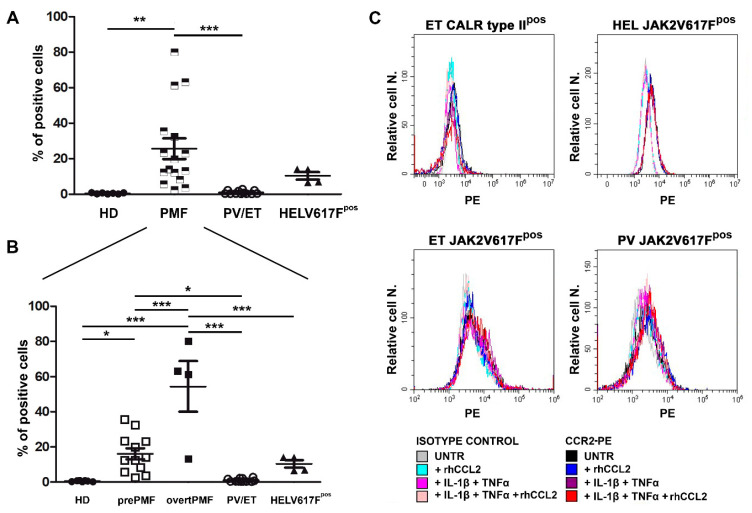
CCR2 expression by normal and MPN CD34+ cells. (**A**) CCR2 expression by flow cytometry (% of positive cells) on immunomagnetically isolated CD34+ cells from n. 7 HD, n. 16 PMF, n. 6 ET, n. 6 PV, and on *JAK2*V617Fpos HEL cells (n. 4 experiments). Data are expressed as mean ± SEM. ** *p* < 0.01, *** *p* < 0.001 by ANOVA followed by Tukey test. (**B**) PMF were split into prePMF (n. 12) and overtPMF (n. 4) and CCR2 expression by flow cytometry (% of positive cells) was compared to HD, PV/ET and HEL. Data are expressed as mean ± SEM. * *p* < 0.05, *** *p* < 0.001 by ANOVA followed by Tukey test. (**C**) Percentage (Relative cell. N.) of CCR2+ cells (freshly isolated CD34+ cells from one *CALR* typeII^pos^ ET patient, upper left panel, one *JAK2*V617F^pos^ ET patient, lower left panel, one *JAK2*V617F^pos^ PV patient, lower right panel, and from *JAK2*V617F^pos^ HEL cells, upper right panel) before (UNTR) and after stimulation with: (i) rhCCL2 100 ng/mL; (ii) IL-1β 1.1 ng/mL and TNFα 10 ng/mL; (iii) rhCCL2, IL-1β and TNFα rhCCL2. Histograms of CCL2-PE specific fluorescence and of isotype-matched irrelevant Ab are reported.

Freshly isolated CD34+ cells were tested for CCR2 expression by flow cytometry. We found that PMF CD34+ cells display >60 fold-higher CCR2 expression as compared to HD, in which, by contrast, it is virtually absent (% of CD34+/CCR2+ cells: 0.42 ± 0.13 in HD vs. 25.65 ± 5.84 in PMF, *p* < 0.01, Figure 3A). Most interestingly, CCR2 expression is a unique characteristic of PMF CD34+ cells, since the receptor is not expressed by other Philadelphia-negative MPN subtypes, namely PV and ET, which share with PMF the same driver mutations (% of CD34+/CCR2+ cells: 0.98 ± 0.27, *p* < 0.001 vs. PMF, Figure 3A).

The inter-individual variability of CCR2 expression detectable among PMF dramatically decreases by stratifying patients according to the disease subtype, with overtPMF significantly overexpressing CCR2 as compared to prePMF (% of CD34+/CCR2+ cells: 54.40 ± 14.36 in overtPMF vs. 16.06 ± 3.14 in prePMF, *p* < 0.001, Figure 3B). Of note, both prePMF and overtPMF significantly overexpress CCR2 as compared to HD and PV/ET (Figure 3B).

The *JAK2*V617F^pos^ cell line HEL has a low expression of CCR2 (% positive cells: 10.34 ± 2.10), with values that are intermediate between the PMF and the PV/ET group (*p* = n.s, Figure 3A) but significantly lower than the overtPMF subtype (*p* < 0.001, Figure 3B).

Overall, these data indicate that CCR2 overexpression (and therefore chemokine susceptibility) is a peculiar characteristic of PMF hematopoietic progenitors.

We subsequently asked whether a pro-inflammatory milieu, in the presence or not of an autocrine loop sustained by CCL2 itself, could induce CCR2 expression in a driver-mutated hematopoietic clone (CD34+ cells from one *CALR* type-II-mutated ET, one *JAK2*V617F-mutated PV and one *CALR* type-I-mutated ET) and in the *JAK2*V617F-mutated cell line HEL. As shown in Figure 3C, CCR2 expression is not triggered by 48 h incubation with IL-1β and TNFα, alone and in combination with rhCCL2, in both MPN CD34+ and HEL cells (Figure 3C).

### 3.5. CCL2/CCR2 Axis Induces an Akt-Dependent Pro-Survival Signal in PMF Hematopoietic Progenitors

**Figure 4:**
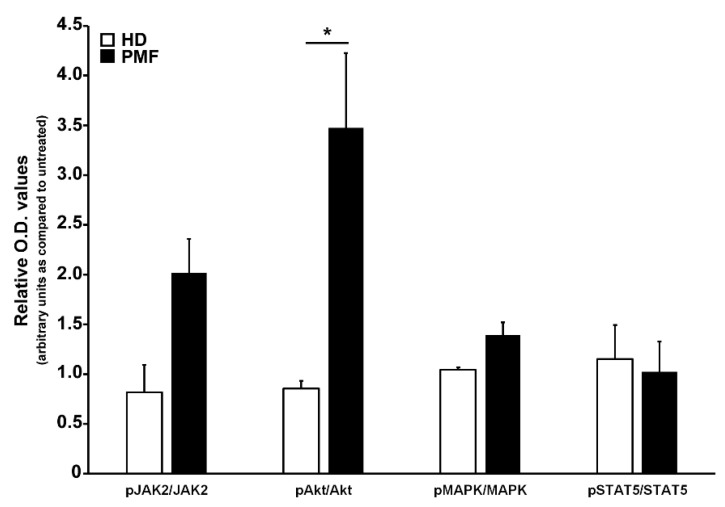
Analysis of signal transduction pathways activated by CCR2 engagement by CCL2 in CD34+ cells. Western blot optical density (O.D.) values obtained from the analysis of phosphorylation levels of JAK2, Akt, MAPK and STAT5 in CD34+ cells from HD (n. 3) and PMF (n. 7) upon 24 h of rhCCL2 stimulation. Relative JAK2/GAPDH, pJAK2/GAPDH, Akt/GAPDH, pAkt/GAPDH, MAPK/GAPDH, pMAPK/GAPDH, STAT5/GAPDH and pSTAT5/GAPDH O.D. values were first determined, and then the phoshorylated/total protein ratio was calculated. Data are represented for both HD and PMF as arbitrary units of untreated samples (mean ± SEM, * *p* < 0.05 by *t*-test).

To ascertain the effects of the CCL2-CCR2 axis in PMF hematopoietic progenitors, we incubated for 24 h PMF CD34+ cells with rhCCL2 to test the activation of downstream signaling cascades related to cell proliferation and differentiation, as well as relevant for MPN, namely JAK/STAT, Akt and MAP-kinase pathways. As control, CCR2-negative CD34+ cells from HD were exposed to the same treatments. As shown in Figure 4, PMF CD34+ cells display significantly increased phosphorylation levels of Akt as compared to control (relative phosphorylated/total protein O.D. values: 3.47 ± 075 vs. 0.86 ± 0.08, data are expressed as fold-changes as compared to untreated samples), indicating that PMF hematopoietic progenitors are an elective target of CCL2, which is capable of inducing, upon CCR2 binding, a prominent activation of the Akt signaling pathway.

### 3.6. CCL2 and CCR2 Expression Are Down-Modulated by Ruxolitinib Therapy

Given the well-established anti-inflammatory and immunomodulatory activity of ruxolitinib [31], we investigated whether the treatment with this drug could affect CCL2 and CCR2 expression in PMF cells.

**Figure 5:**
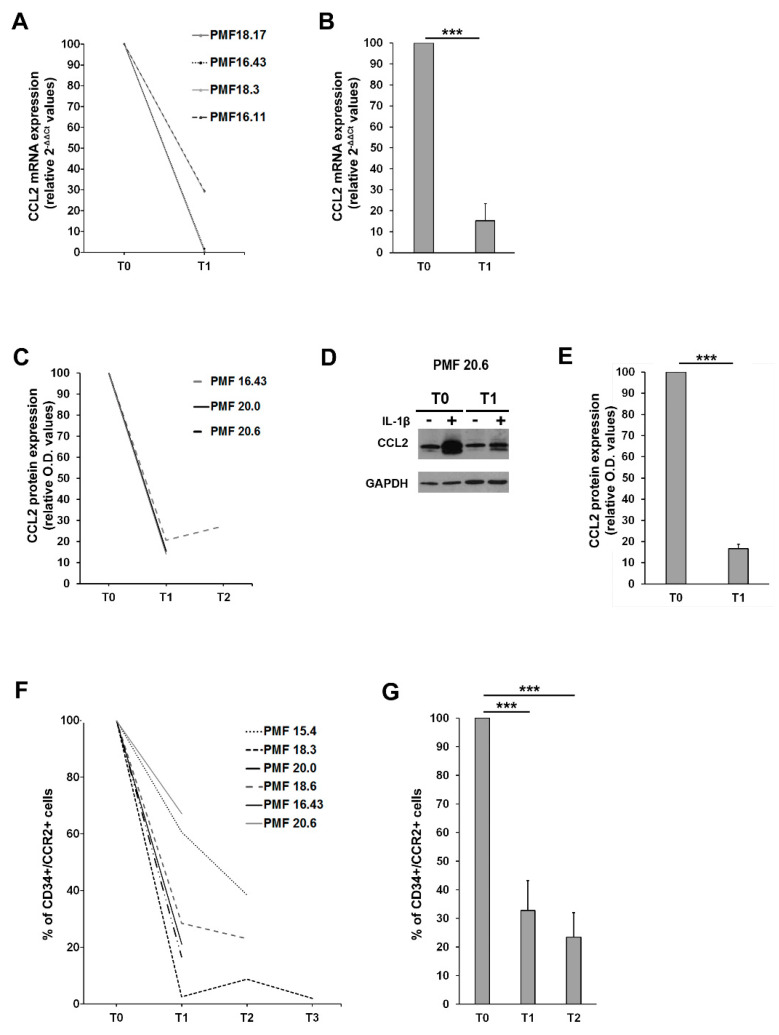
Effects of ruxolitinib on CCL2 and CCR2 expression (**A**) Fold-decrease curves (2-[(CT CCL2-CT GAPDH)IL1-β − (CT CCL2-CT GAPDH)untr, relative 2-ΔΔCT values) of CCL2 mRNA expression upon ex vivo IL1-β stimulation of MNCs from 4 PMF patients before (T0) and after 1 month (T1) of ruxolitinib therapy. T0 values were set as reference. (**B**) Fold-decrease (2-[(CT CCL2-CT GAPDH)IL1-β − (CT CCL2-CT GAPDH)untr, relative 2-ΔΔCT values) of CCL2 mRNA expression upon ex vivo IL1-β stimulation of MNCs from 4 PMF patients before (T0) and after 1 month (T1) of ruxolitinib therapy. Data are expressed ad mean ± SEM as compared to T0 (*** *p* < 0.001 by *t*-test). (**C**) Fold-decrease curves [(O.D.CCL2/O.D.GAPDH)IL-1β/[(O.D.CCL2/O.D.GAPDH)UNTR, relative western blot optical density (O.D.) values] of CCL2 protein expression upon ex vivo IL1-β stimulation of MNCs from 3 PMF patients before (T0) and after 1 (T1) and 3 months (T2) of ruxolitinib therapy. T0 values are set as reference. (**D**) Representative Western blot showing CCL2 protein expression in MNCs from the PMF#20.6, at a basal state (−) and upon IL1-β stimulation (+), before (T0) and after 1 month of ruxolitinib (T1). (**E**) Fold-decrease [(O.D.CCL2/O.D.GAPDH)IL 1β/[(O.D.CCL2/O.D.GAPDH)UNTR, relative western blot optical density (O.D.) values] of CCL2 protein expression upon ex vivo IL1-β stimulation of MNCs from PMF 3 patients before (T0) and after 1 month (T1) of ruxolitinib therapy. Data are expressed ad mean ± SEM as compared to T0 (*** *p* < 0.001 by *t*-test). (**F**) Fold-decrease curves of CCR2 expression assessed by flow cytometry (% of positive cells) on immunomagnetically isolated CD34+ cells from 6 PMF before (T0) and after 1 (T1), 3 (T2) and 9 (T3) months of ruxolitinib. T0 values are set as reference. (**G**) Fold-decrease of CD34+/CCR2+ cells after 1 (T1) and 3 months (T2) of ruxolitinib therapy in 6 PMF. Data are expressed as mean ± SEM as compared to T0 (*** *p* < 0.001 by one-way ANOVA followed by Tuckey test).

CCL2 expression was assessed in MNCs from PMF patients before (T0) and after ruxolitinib start (T1 = 1 month, T2 = 3 months). MNCs were stimulated ex vivo with 1.1 ng/mL of IL-1β for 20 h, as described above, and CCL2 expression was assessed at both mRNA and protein level. MNCs from on-drug patients display a significantly reduced expression of CCL2 mRNA (Figure 5A,B) and protein (Figure 5C–E) after IL-1β stimulation as compared to T0. Overall, after only one month of therapy, we could detect a >80% reduction of CCL2 mRNA (*p* < 0.001 vs. T0) and protein (*p* < 0.001 vs. T0) expression in PMF MNCs exposed to appropriate inflammatory stimuli (Figure 5B,E data are expressed as fold-decrease of relative 2^−ΔΔCT^ and O.D. values). Although the sample size does not allow us to draw statistical considerations, it is worth noting that G/G PMF cells are the ones experiencing the most pronounced reduction in CCL2 expression (both mRNA and protein) on ruxolitinib treatment (Supplementary Materials Figure S1), potentially identifying homozygous patients as the most sensitive category to immunomodulatory drugs.

We then assessed CCR2 expression on PMF CD34+ before (T0) and after ruxolitinib start (T1 = 1 month, T2 = 3 months and T3 = 9 months), detecting a significant and stable reduction of the surface expression of the chemokine receptor (>60% reduction at T1 and >70% reduction at T2, *p* < 0.001 vs. T0 in both cases, data are expressed as fold-decrease of the percentage of CD34+/CCR2+ cells) (Figure 5F,G). Collectively, these data indicate that ruxolitinib therapy is able to turn off the CCL2-CCR2 chemokine system in PMF.

## 4. Discussion

PMF is a clonal disorder of the hematopoietic stem cell with a strong pro-inflammatory component. In fact, although its pathogenesis is cell-intrinsic and triggered by acquired somatic mutations in specific myeloid genes, cell-extrinsic effects exerted by the malignant clone via inflammatory mediators result in both local (bone marrow fibrosis) and systemic (constitutional symptoms, thrombosis) complications [6]. Moreover, chronic inflammation itself fosters clonal evolution, in a self-perpetuating vicious cycle described by Hasselbalch as “the human inflammation model for cancer development” [5]. Host genetic factors such as SNPs began to emerge as important players in determining not only the individual susceptibility to MPN onset [32], but also in tuning the disease phenotype [11,25,33,34] and response to therapy [35]. Given this scenario, host genetic variations affecting individual inflammatory states such as SNPs in cyto/chemokine genes are of utmost interest in the context of MPNs. In this study we focused on the rs1024611 SNP of *CCL2,* typified by an A to G substitution at position -2518 of the *CCL2* 5′-flanking region and capable of enhancing the transcriptional activity of the distal regulatory region upon inflammatory stimulus [21,22,23]. Over the years, this SNP has been associated to several chronic inflammatory conditions, including cancer [36].

In a cohort of 773 well-characterized PMF, we investigated the contribution of this SNP to demographical, clinical and biological characteristics and its impact on disease outcome. While the overall PMF population displayed similar genotypic and allelic frequencies of local controls, male subjects carrying the homozygous genotype G/G had a higher risk of PMF than wild type or heterozygous genotypes, indicating a genetic background interplay between gender and *CCL2* variants. Indeed, the fact that the homozygosity for the SNP qualifies as a host predisposition factor for the disease only in the male cohort may hint at the presence of a sex-related background that elicits (males) or mitigates (females) the effect of the SNP. In this context, several studies have identified a sex-specific transcriptome and methylome, which acts independently from the well-characterized phenomenon of X-chromosome inactivation, suggesting that sexual dimorphism also occurs at the epigenetic level [37].

The analysis of the patient dataset documented that the homozygosity for the -2518G allele variant of the *CCL2* gene is an independent prognostic factor for reduced survival in PMF. These results, obtained from a robust cohort of patients with a full hematological, genetic and biological characterization, suggest that the rs1024611 G/G genotype may represent a high-risk variant and a novel host genetic determinant of shortened survival in PMF. The fact that G/G PMF displayed, at the time of diagnosis, similar hematologic characteristics of A/A and A/G suggests that, given the same clinical phenotype at the time of disease onset, patients carrying a homozygous genotype for the rs1024611 SNP are expected to have a shortened survival as compared to heterozygous and wild types. Of note, the G/G cohort was not significantly enriched in adverse molecular features, such as the triple-negativity or HMR mutations. An apparent limitation of this finding might be identified in the lack of an independent validation set, which, however, is overcome by the subsequent mechanistic connection of the rs1024611 to the biology of the disease. In fact, these clinical observations prompted us to investigate the functional role of the SNP in PMF cells and the effects of the CCL2/CCR2 axis’ activation.

We demonstrated that PMF cells carrying a homozygous rs1024611 genotype are the highest chemokine producers as compared to the other genotypes, validating, in the MPN setting, the dose-dependent effect of the SNP described in healthy individuals in the seminal paper by Rovin et al. [21]. This supports the choice of the recessive genetic model for comparisons in the PMF cohort, enabling at the same time the definition of G/G PMF as the high-risk genotype.

To ascertain the potential cellular targets of CCL2 in PMF, we found that PMF hematopoietic progenitors selectively overexpress CCR2 as compared not only to HD but also to other MPN subtypes, such as ET and PV. Of note, we also demonstrated that the pro-inflammatory milieu, in the presence or not of an autocrine loop, is not capable of inducing CCR2 expression in primary CD34+ cells from PV/ET (carrying driver mutations) or in the *JAK2*V617F^pos^ HEL cell line. With the limits related to the low number of PV/ET samples utilized for this purpose, we can hypothesize that other mechanisms, possibly related to *CCR2* transcriptional regulation, that act independently from inflammatory triggers, regulate the unique and selective expression of CCR2 on PMF CD34+ cells. Interestingly, overtPMF significantly overexpress CCR2 as compared to prePMF, suggesting that CCR2 expression on CD34+ circulating cells can be envisioned as a marker of bone marrow fibrosis (irrespective of rs1024611 SNP). Further studies on a larger patient dataset are needed to confirm this hypothesis.

CCR2 engagement by its ligand significantly increases the activation state of the Akt signaling pathway in PMF cells, while CCR2-negative CD34+ cells do not display CCL2-mediated phosphorylation of Akt or of other pro-proliferative signal transduction pathways. Selective Akt pathway activation is consistent with what was described in solid cancers such as breast, ovarian and prostate [38,39,40]. Therefore, Akt is the main signaling pathway conveying a CCL2/CCR2-dependent proliferative signal in PMF hematopoietic progenitors, boosting pro-survival signals induced by driver mutations.

We then asked whether immunomodulatory therapy could interfere with the CCL2/CCR2 signaling axis, focusing on ruxolitinib, which currently represents the therapeutic standard for symptomatic intermediate- to high-risk MF. Ruxolitinib excels by effectively reducing splenomegaly and the constitutional symptom burden, the latter being the main clinical manifestation of the disease-related cytokine storm [41]. We proved that ruxolitinib is able to effectively down-regulate CCR2 expression on PMF CD34+ cells and de-sensitize PMF cells to IL-1β-dependent pro-inflammatory stimulus, reducing their capability to express CCL2 mRNA and protein.

**Figure 6:**
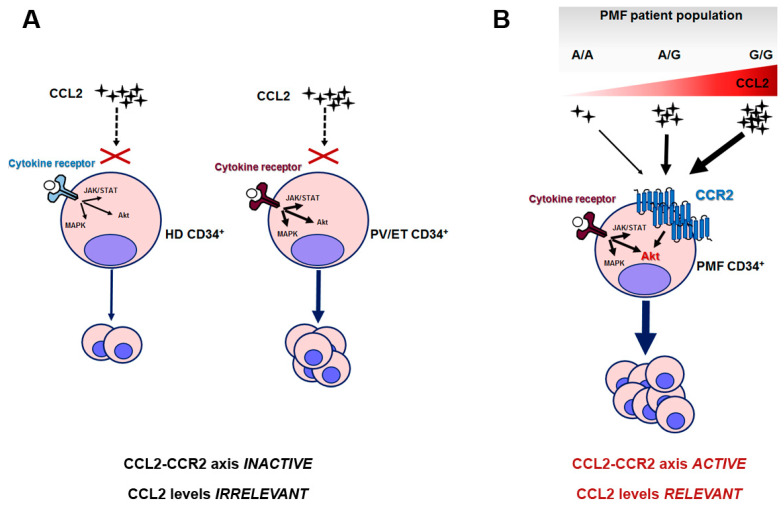
Proposed mechanistic scheme of the relevance of the CCL2/CCR2 axis in PMF and the related consequences of rs1024611 SNP. (**A**) HD and PV/ET CD34+ cells do not express CCR2, and pro-proliferative signals are transduced by cytokine-receptor-mediated downstream signaling, i.e., JAK/STAT, MAP-kinase and PI3K/Akt pathways; in ET and PV the intracellular signaling is constitutively active due to the presence of somatic driver mutation(s) and accounts for myeloproliferation. (**B**) PMF CD34+ cells uniquely express CCR2, which signals mainly via the Akt pathway. CCR2-downstream signaling potentiates cytokine-receptor signaling, also constitutively activated, in this case, by somatic driver mutation(s). In this setting, CCL2 levels become extremely relevant: due to the dose-dependent effects of the G allele on CCL2 production, A/A and A/G may result in a mild disease phenotype, while G/G subjects, who are genetically predisposed to produce the highest chemokine levels, will result in a more aggressive disease. In other terms, when the PMF neoplastic clone arises in a G/G host genetic background, it will lead to a disease with worse outcome.

Overall, we can envision a scenario in which the PMF clone is uniquely sensitive to CCL2 levels because of its elective expression of CCR2. When this clone arises (due to the occurrence of driver mutations) in a host genetic background characterized by CCL2 overproduction, such as in G/G individuals, this endorses a pro-survival signal in PMF hematopoietic progenitors, which can explain the worse outcome of homozygous patients. In healthy subjects and PV or ET, instead, in which CCL2 levels are likely modulated by the rs1024611 SNP in a similar fashion, chemokine levels are irrelevant for CD34+ proliferation, since HD and PV/ET CD34+ do not express (or express at very low levels) CCR2 (Figure 6).

Taken together, our data provide the biologic rationale for considering a novel therapeutic strategy in PMF based on CCL2/CCR2 axis inhibition. In this context, small-molecule CCR2 inhibitors and antibodies against CCR2 or CCL2 have been developed to interfere with CCL2-CCR2 signaling. These compounds, tested in both phase-1 and phase-2 clinical trials in solid tumor and inflammatory diseases, demonstrated a good safety and tolerability profile [42,43,44]. Moreover, in a perspective of tailored therapy, Gilbert and co-workers reported that MLN1202, a highly specific humanized monoclonal antibody that interacts with CCR2 and inhibits CCL2 binding, induced a significant reduction of high-sensitivity C-reactive protein in atherosclerotic patients, which was more pronounced in those carrying the A/G or G/G rs1024611 genotypes [45].

## 5. Conclusions

Our data first describe the role of the CCL2/CCR2 chemokine system in PMF, and pave the way for rs1024611 SNP genotyping as a potential novel strategy to risk-stratify patients—not influenced, as a germline variant, by clonal evolution or therapy. We also proved that the CCL2/CCR2 chemokine system is selectively activated in PMF, boosting pro-survival signals induced by driver mutations, laying the ground for further studies on CCL2/CCR2 axis inhibition as a novel therapeutic strategy in this disease. Finally, we provided a novel mechanism underlying the anti-inflammatory effects of ruxolitinib, via the simultaneous down-regulation of CCL2 production and CCR2 expression in PMF cells.

## Figures and Tables

**Table 1:** Demographic features of PMF (at the time of diagnosis) and CTRL, stratified according to the *CCL2* rs1024611 SNP genotype.

		*CCL2* rs1024611 Genotype			*p-*Value OR, [95% CI]
	N.	A/A	A/G	G/G	(G/G vs. A/A+A/G)
**PMF Demographics**					
**PMF, tot** N, %	773	430 (55.6%)	286 (37.0%)	57 (7.4%)	*p* = 0.09 vs. CTRL tot.
**PMF, males** N, %	464	247 (53.2%)	174 (37.5%)	43 (9.3%)	***p* = 0.014 vs. PMF females** **OR, 2.15 [1.15–4.00]** ***p* = 0.015 vs. CTRL tot.** **OR, 2.10 [1.14–3.84]** ***p* = 0.022 vs. CTRL males** **OR, 2.52 [1.11–5.72]**
**PMF, females** N, %	309	183 (59.2%)	112 (36.2)	14 (4.5%)	
**PMF, tot** Age yrs, median (range)	773	50 (6–80)	51 (16–81)	54 (18–83)	***p* = 0.048**
**CTRL Demographics**					
**CTRL, tot** N, %	323	186 (57.6%)	122 (37.8%)	15 (4.6%)	
**CTRL, males** N, %	180	101 (56.1%)	72(40.0%)	7 (3.9%)	*p* = 0.5 vs. CTRL females
**CTRL, females** N, %	143	85 (59.4%)	50 (35.0%)	8 (5.6%)	
**CTRL, tot** Age, yrs median (range)	323	63 (31–86)	61 (28–85)	64 (52–75)	*p* = 0.9

(A/A = wild type; A/G = heterozygous; G/G homozygous). Statistically significant correlations are highlighted in **bold**.

## Data Availability

The data presented in this study are available on request from the corresponding author.

## Supplementary Materials

The following are available online at https://www.mdpi.com/article/10.3390/cancers13112552/s1, supplementary materials and methods, Table S1: Clinical and laboratory features of PMF patients at the time of diagnosis stratified according to the CCL2 rs1024611 genotype, Table S2: Univariate and multivariate analysis of prognostic factors for survival in PMF. Figure S1. Effects of ruxolitinib on CCL2 expression in PMF cells according to the rs1024611 genotype.
