# Supplementary material for: Impact of the rs1024611 Polymorphism of CCL2 on the Pathophysiology and Outcome of Primary Myelofibrosis
Source: Cancers (Basel). 2021 May 22;13(11):2552. doi: 10.3390/cancers13112552 (PMC8196972; doi:10.3390/cancers13112552)
Supplement: Supplementary file 1 [file cancers-13-02552-s001.zip › cancers-1222146-supplementary.pdf]

cancers

MDPI

Supplementary Materials: Impact of the rs1024611 Polymorphism of *CCL2* on the Pathophysiology and Outcome of Primary Myelofibrosis

Elena Masselli, Cecilia Carubbi, Giulia Pozzi, Antonio Percesepe, Rita Campanelli, Laura Villani, Giuliana Gobbi, Sabrina Bonomini, Giovanni Roti, Vittorio Rosti, Margherita Massa, Giovanni Barosi and Marco Vitale

Supplemental Materials and Methods.

Clinical and biological characteristics of study and control populations

Four-hundred and sixty four out of 773 PMF patients (60%) were males, with a median age of 52 years (range, 6-83 years). Bone marrow biopsy at diagnosis was available in 768 cases; according with the WHO classification, n. 428/773 (55.7%) had a diagnosis of pre-fibrotic myelofibrosis (pre-PMF). IPSS risk distribution was 416/684 (60.8%) low, 112/684 (16.4%) intermediate-1, 92/684 (13.4%) intermediate-2, and 64/684 (9.3%) high. Driver mutations were tested in 757 (98%) patients and occurred as follows: 500 (66%) harbored *JAK2*V617F mutation (of which n. 194, 38.8% at the homozygous state), 145 (20.3%) had *CALR* mutations, 61 (8.1%) were triple negative and 42 (5.5%) had *MPL* mutations. Two-hundred and twenty-five patients were screened for HMR mutations and 50 (22.2%) fell into HMR category (Supplemental Table 1)

After a median follow-up of 77 months, 86 on 773 patients (11.1%) received ASCT and 191 patients (24.7%) died, producing a 10-year survival of 75%. One hundred and thirty-eight patients (17.8%) had documented blast transformation (BT), with a 10-year BT free survival of 80%. Other causes of death were mainly represented by PMF-related complications (37% of cases, including bone marrow failure, cachexia, infections, and multi-organ-failure); ASCT-related complications accounted for 15% of deaths and thrombosis/hemorrhage for 18% of cases.

One-hundred and eighty out of 323 CTRL were males (55.7%) and their median age was 63 years (range 28-86 years).

NGS analysis

NGS analysis for target myeloid genes was performed by Myeloid Solutions Panel (SOPHiA Genetics, Saint Sulpice, Switzerland and Arrow Diagnostics). 200 ng of DNA was utilized for libraries preparation and pair-end sequencing on a MiSeq (Illumina, San Diego, CA, USA) using Reagent Kit V2 500 cycles cartridge, according to manufacturer´s instructions. Alignment, base calling and variant annotation were performed with SOPHiA DDM software and interrogating available databases (such as NCBI, COSMIC, GNOMAD, CLINVAR, ExAC).

Table S1. Clinical and laboratory features of PMF patients at the time of diagnosis stratified according to the *CCL2* rs1024611 genotype (A/A=wild type; A/G=heterozygous; G/G=homozygous).

			***CCL2* rs1024611 genotype**			***P* value**
	**N. of cases evaluated**	**All patients**	**A/A**	**A/G**	**G/G**	**(G/G *vs.* A/A+A/G)**
**Clinical characteristics**						
**Hb** mean (range), g/dL	768	12.7 (3.0-22.0)	12.9 (4.0-22.0)	12.6 (3.0-22.0)	12.6 (5.0-20.0)	0.71

**Pts with Hb <10 g/dL** N. (%)		142 (18.5)	78 (18.3)	50 (17.7)	14 (24.5)	0.22
**WBC**[a] mean (range), $x10^9$/L	761	10.0 (1.8-64.3)	9.9 (1.9-64.3)	10.2 (1.8-45.4)	10.2 (2.6-50.9)	0.81
**Pts with WBC >12$x10^9$/L** N. (%)		172 (22.6)	91 (21.6)	69 (24.5)	12 (21.0)	0.77
**PLT** mean (range), $x10^9$/L	764	509 (21-2926)	523 (22-2926)	488 (21-2000)	508 (37-1783)	0.98
**Pts with PLT<150 $x10^9$/L**, N. (%)		113 (14.8)	56 (13.1%)	46 (16.4%)	11 (19.6%)	0.29
**Spleen index**[b] mean (range), cm^2	761	151.0 (70-1200)	150 (70-850)	154 (89-1200)	143 (90-486)	0.57
**Pts with spleen index**[b] **>150 cm2** N. (%)		213 (28.0)	110 (26.1)	86 (30.5)	17 (29.8)	0.75
**LDH** mean (range), mU/mL, x ULN	429	1.66 (0.19-8.48)	1.58 (0.41-8.48)	1.70 (0.19-7.60)	2.02 (0.67-7.38)	0.07
**Monocytes,** mean (range), $x10^9$/L	411	612 (23-3600)	594 (23-3360)	657 (50-3600)	529 (37-1543)	0.36
Splanchnic vein thrombosis at dx N. (%)	773	73 (9.4)	42 (9.8)	26 (9.1)	5 (8.8)	0.86
**Peripheral blood CD34$^+$ cells** mean (range), $x10^6$/L	355	64.0 (0.4-1601)	53.9 (0.4-1152)	83.1 (0.9-1601)	64.8 (0.4-1601)	0.71
**Serum cholesterol** mean (range)	377	158 (51-307)	163 (69-304)	135 (64-307)	148 (51-227)	0.20
**Inflammatory biomarkers**						
**hs-CRP** mean (range), ng/mL	222	0.70 (0.01-12.6)	0.68 (0.01-12.6)	0.74 (0.02-7.9)	0.75 (0.02-4.38)	0.90
**Pts with hs-CRP >0.3 ng/mL** N. (%)		88 (39.6)	52 (37.9)	27 (38.0)	9 (64.3)	0.05
			**Mutations**			
***JAK*2V617F positive** N. (%)	757	500 (66.0)	277 (55.4)	185 (37.0)	38 (7.6)	0.77
***JAK*2V617F negative** N. (%)		257 (34.0)	142 (55.3)	97 (37.7)	18 (7.0)	
***JAK*2V617F positive, heterozygous** N. (%)	500	306 (61.2)	174 (56.9)	109 (35.6)	23 (7.5)	1.00
***JAK*2V617F positive, homozygous** N. (%)		194 (38.8)	103 (54.1)	76 (39.2)	15 (7.7)	
***CALR* positive (type 1 or 2)** N. (%)	762	157 (20.6)	85 (54.1)	60 (38.2)	12 (7.7)	0.87

***CALR* negative**<br>N. (%)		605 (79.4)	337 (55.7)	224 (37.0)	44 (7.3)	
***MPL*W515 positive**<br>N. (%)	761	42 (5.5)	25 (59.5)	16 (38.1)	1 (2.4)	0.20
***MPL*W515 negative**<br>N. (%)		719 (94.5)	396 (55.1)	268 (37.3)	55 (7.6)	
**Triple-negative**<br>N. (%)	757	61 (8.1)	34 (55.7)	22 (36.1)	5 (8.2)	0.80
**Non triple-negative**<br>N. (%)		696 (91.9)	385 (51.0)	260 (34.3)	51 (6.7)	
**≥ HMR category[c]**	225	50 (22.2)	26 (52.0)	17 (34.0)	7 (14.0)	0.39
**Non-HMR category[c]**		175 (77.8)	107 (61.1)	51 (29.1)	17 (9.8)	

Statistically significant correlations are highlighted in bold. [a] White-blood cell count (WBC) was corrected for the number of circulating erythroblasts. [b] Spleen index is the product of the longitudinal by the transverse spleen axis, the latter defined as the maximal width of the organ. [c] defined as presence of at least one mutated gene among ASXL1, EZH2, SRSF2, IDH1/2[8]. Abbreviations: Hb: hemoglobin, hs-CRP: high-sensitivity C reactive protein, LDH: Lactate dehydrogenase, PLT: Platelet count, Pts: patients, WBC: white blood cell count.

Table S2. Univariate and multivariate analysis of prognostic factors for survival in PMF.

		**Univariate analysis**		**Multivariate analysis**	
**Variable**	**N. at risk**	**HR (95% CI)**	**P**	**HR (95% CI)**	**P**
**Age (diagnosis)**<br>**> 65 y**	122	6.66 (4.76-10)	<0.001	4.11 (3.03-8.25)	<0.001
**Constitutional symptoms**	154	5.88 (4.16-8.33)	<0.001	2.60 (1.75-3.85)	<0.001
**Hb**<br>**< 100 g/L**	142	4.54 (3.83-6.25)	<0.001	2.19 1.85-4.00)	<0.001
**WBC**<br>**> 25 x10^9/L**	28	5.00 (2.70-9.09)	<0.001	3.88 (1.82-5.66)	<0.001
**Peripheral blasts**<br>**≥1**	68	5.55 (3.45-9.09)	<0.001	3.17 (1.76-5.69)	<0.001
***CCL2* rs1024611**<br>**G/G genotype**	57	1.69 (1.05-2.70)	0.032	1.65 (1.01-2.70)	0.039

Analysis was performed in 773 PMF and included IPSS parameters and CCL2 rs1024611 G/G genotype. Abbreviations: Hb: hemoglobin, WBC: white blood cell count.

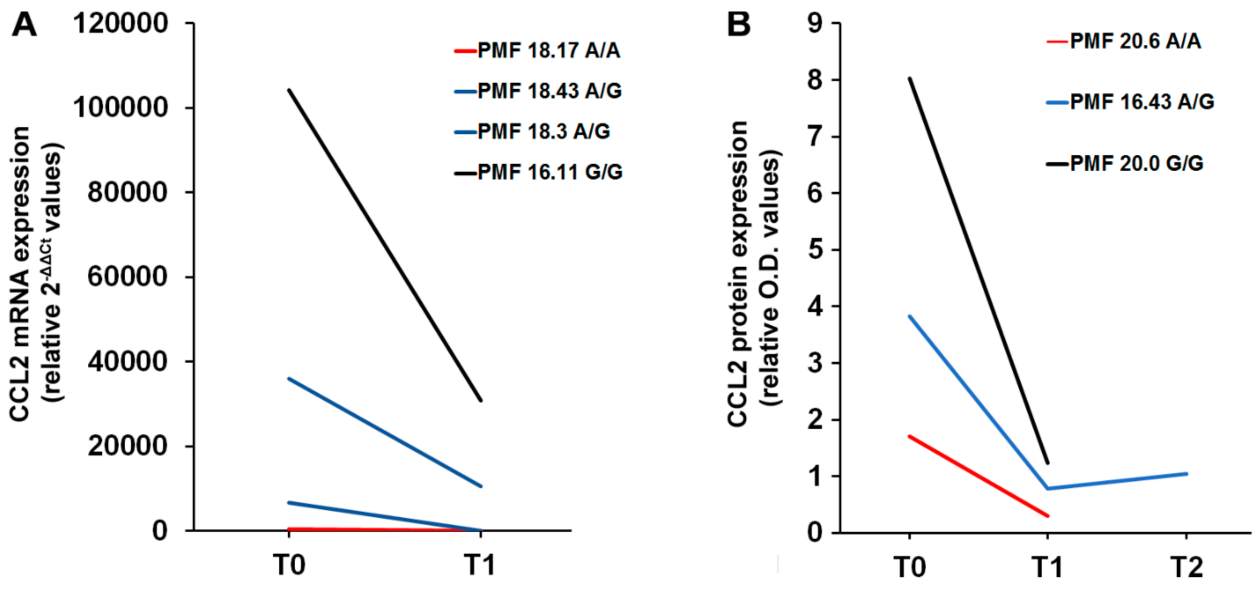

Figure S1. Effects of ruxolitinib on CCL2 expression in PMF cells according to the rs1024611 genotype. (**A**) Fold-decrease curves (2-[(CT CCL2-CT GAPDH)IL1-β − (CT CCL2-CT GAPDH)untr, relative 2-ΔΔCT values) of CCL2 mRNA expression upon ex-vivo IL1-β stimulation of MNCs from 4 PMF patients before (T0) and after 1 month (T1) of ruxolitinib therapy, stratified according to the rs1024611 genotype. (**B**) (Fold-decrease curves [(O.D.CCL2/O.D.GAPDH)IL-1β/[(O.D.CCL2/O.D.GAPDH)UNTR, relative western blot optical density (O.D.) values] of CCL2 protein expression upon ex-vivo IL1-β stimulation of MNCs from 3 PMF patients before (T0) and after 1 (T1) and 3 month (T2) of ruxolitinib therapy, stratified according to the *CCL2* rs1024611 genotype.
